# Acceleration of Atmospheric Freeze‐Drying of Food: Methods, Implications, Commercialization, and Future Directions

**DOI:** 10.1111/1541-4337.70432

**Published:** 2026-02-23

**Authors:** Nikita S. Bhatkar, Anarghya Ananda Murthy, Siew Young Quek, Meng Wai Woo

**Affiliations:** ^1^ Department of Chemical and Materials Engineering, Faculty of Engineering University of Auckland Auckland New Zealand; ^2^ Department of Mechanical & Mechatronics Engineering, Faculty of Engineering University of Auckland Auckland New Zealand; ^3^ Food Science, School of Chemical Sciences University of Auckland Auckland New Zealand

**Keywords:** atmospheric freeze‐drying, drying rate, intensification, vacuum freeze‐drying

## Abstract

Atmospheric freeze‐drying (AFD) is an emerging and cost‐effective alternative for drying food and pharmaceutical ingredients, offering lower investment and operational costs compared with vacuum freeze‐drying (VFD). However, industrial adoption is limited due to the significantly longer time required, resulting from consistently lower drying rates. This has led to increasing interest in accelerating AFD through various strategies. Several approaches have been explored, which can be classified as thermal methods, enhanced vapor flow, reduction in internal resistance, and other pretreatments. While these methods show some degree of acceleration, understanding their implications and limitations is essential for their adoption in AFD. Despite ongoing research on acceleration methods, the analysis of the commercial presence of AFD suggests a significant gap between research progress and the applicability of these methods. This review offers insight into these acceleration methods and provides some future considerations for research to enhance the viability of the process.

## 1 Introduction

Freeze‐drying as a process can be traced back to prehistoric times. However, it was in 1905 that the method was first reported for drying animal tissue (Varshney and Singh 2015). The idea of removing water at a reduced temperature from the sample through sublimation was relatively nascent, and this fascinated various studies in this direction around the concept. The most promising feature of this drying method is the sublimation of solid water at low‐temperature operation, which enables it to preserve heat‐sensitive compounds and minimize changes in the quality characteristics of the sample. Moreover, freeze‐dried food and pharmaceutical ingredients are superior to any other drying method, with minimal effect on the sample quality (Abla and Mehanna 2022; Harguindeguy and Fissore 2020). However, the superior quality of the product is ensured at the cost of longer processing time, high initial capital investments, and higher operating costs relative to other hot‐air drying methods. Many attempts have been made to make it viable to reduce the operating cost by utilizing other advanced conjugation methods, such as microwave (MW), infrared (IR), ultrasound (US), and so on. Despite these methods successfully reducing operating costs and maintaining substantial product quality standards, a higher initial investment and operating cost for maintaining a strong vacuum in the system still persist. Nevertheless, the freeze‐drying industry has accepted this limitation.

While the industry has long identified that the sublimation of water requires a vacuum, in essence, the process can occur at ambient pressure. A case in point is the drying of frozen laundry hung out in the winter (Hua et al. 2010). At the scientific front, in 1959, Dr. Harold Meryman formally proposed that water sublimation can occur at atmospheric pressure and that the process is driven by the vapor pressure gradient in the system (Claussen, Ustad, et al. 2007). In the following years, various preliminary studies were conducted by Lewis and Woodward (Lewin and Mateles 1962; Woodward 1963). These studies revealed that atmospheric freeze‐drying (AFD) could potentially reduce the initial capital investment and the operation cost, as there is no need to maintain a vacuum environment; however, the drying rates of AFD are consistently found to be lower than vacuum freeze‐drying (VFD) (Reyes, Vega, et al. 2010; Donsì et al. 2001).

One could argue that this could be due to the lower vapor transport resistance in VFD and the absence of water vapor molecules in the vacuum environment. In contrast, water vapor transport in AFD, to a certain extent, is impeded by the presence of vapor (Chen et al. 2020). Despite the relatively higher thermal conductivity at ambient pressure (AFD) (Hu et al. 2025), this mass transfer resistance seemed to be the limiting rate factor for AFD compared to VFD.

Another aspect to consider is the temperature to which the product can potentially be exposed. In VFD, the plate temperature can be ramped up to higher temperatures, especially toward the later stages of drying, where the temperature in AFD is limited to air temperatures below the freezing point (−30°C to less than 0°C). This temperature difference also enhances the drying rate of VFD.

Given this main limitation, over the years, many reports of work have been done to accelerate the AFD process to improve its drying rate. Considering these, there are studies where the particle size of the sample was reduced, and the drying rates were enhanced. Over the years, many other methods, such as temperature ramping, spray freeze‐drying (SFD), fluidization, spouted bed fluidization, and the use of adsorbents, have been explored to accelerate AFD. An attempt to improve the drying rate with an impinging jet was recently explored (Y. Xu et al. 2024). Most of these methods have been quite promising in increasing the drying rate. However, some of them still have some associated drawbacks. Previous review articles on AFD have addressed the fundamental principles of AFD along with an extensive coverage of mathematical modeling and a broad coverage of the technologies complementary to the AFD process (Azizpour et al. 2025; Naliyadhara and Trujillo 2025). The current review provides a more streamlined mechanistic critique of the techniques specific to enhancing the drying rate (drying time reduction) of the AFD process. This is the current bottleneck of the AFD process. A mechanistic framework is introduced in this review to help readers evaluate the proper implementation, advantages, and limitations of each technique. Along that line, past reviews also did not evaluate the current state of commercial uptake of the AFD process with its associated complementary enhancing technology. This review aims to address this gap by surveying the reported patents on AFD. It will be revealed later on that there is a significant gap between research and actual commercial applicability. By doing so, it aims to establish a clear framework for individuals from both research and industrial backgrounds in advancing the AFD‐related process.

## 2 AFD

### 2.1 Historic Evolution of AFD and Acceleration Methods

AFD is a precedent phenomenon even before it was formally reported in 1959. The existence could be traced back to the everyday practice of drying laundry in cold winters in Asian countries, where frozen laundry was dried by sublimating water from frozen clothes at atmospheric pressure in the presence of cold, dry air. The formal pioneering work on AFD was reported by Harold Meryman in 1960, who demonstrated drying of frozen mammalian specimens at −30°C using dry air (Meryman 1960). Subsequently, proof‐of‐concept studies were carried out by Lewin and Mateles (1962) and Woodward (1963) in the next decade. Alongside it was reported that the drying time for AFD is slower than that of VFD. During the same period, AFD gained popularity as a preservation technique for vacuum‐sensitive bacterial cells, as it enhanced the viability of the cells during drying (Wagman and Weneck 1963). Likewise, AFD was used to freeze‐dry the insects (Flaschka and Floyd 1969).

In the following decade, more advances were made. A detailed heat and mass transfer analysis of AFD was conducted by Heldmann and Honer (Hohner 1970). Sonic energy was first applied to intensify the AFD process for tea and coffee extracts (Moy and DiMarco 1970). In the upcoming years, drying of various food and pharmaceutical ingredients was studied, and various acceleration methods were employed. Gibert introduced the concept of immersing food in a fluidised bed of adsorbent to accelerate the process, followed by the exploration of simple lyophilization using the adsorbed medium.

In the 21st century, the innovations have mostly focused on integrating AFD with heat pumps and related designs. Later, S.M.A. Rahman introduced methods such as vortex tube and vibrio fluidization with adsorbent to intensify AFD. Recently, other extrinsic factors, such as air‐guided models and impinging jets, have been used to accelerate AFD.

### 2.2 Comparison With VFD

The freeze‐drying at atmospheric pressure involves sublimation of ice at low temperatures and ambient pressure. In the case of VFD, the process is based on the principle of lowering the pressure in the surroundings to a point lower than the triple point of water and providing the heat for the water in the frozen state to escape as vapor without converting to a liquid state. The moisture is then liberated from the product because of the pressure gradient, which facilitates drying. AFD also follows the same principle as that of VFD, where the driving force is the vapor pressure gradient. However, unlike VFD, the prevailing conditions in AFD are above the triple point of water, but the temperature is maintained low enough for the moisture to exist in the solid state.

The drying medium (air) plays the most crucial role in AFD, which is devoid of moisture, creating a vapor pressure gradient between the ice and cold air. The air temperature is essentially kept below the freezing point of the food to avoid melting at ambient pressure. This is very dissimilar to VFD, where the temperature in the chamber may be maintained close to ambient conditions, which in turn gives a higher vapor pressure gradient.

**FIGURE 1:**
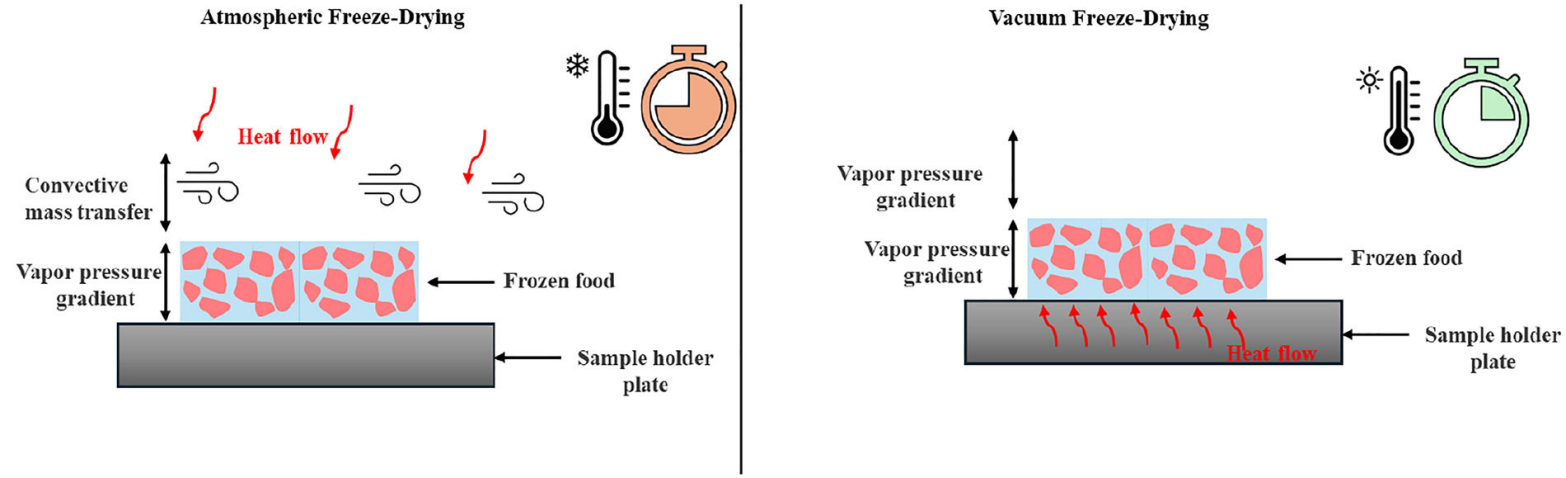
Illustration for comparison of AFD and VFD.

In both AFD and VFD, the vapor pressure gradient is responsible for the transfer of moisture from the interior of the food to the surface. Once the moisture reaches the surface, it is carried from the surface to the surroundings through the convective mode by the continuously circulated cold, dry air. Meanwhile, in VFD, the process takes place under vacuum conditions. In the presence of a drying medium in AFD, the heat transfer coefficient is relatively high compared to VFD. Though there is a significant difference (20–40 times) in the heat transfer coefficient in VFD and AFD, the higher temperature prevailing in the VFD chamber imparts a driving force for drying. A higher temperature in VFD results in a higher vapor pressure gradient between the interior of the food and the surface, which, to some extent, increases the thermal conductivity of food and the mass transfer coefficient of moisture. This implies that moisture removal from the food's interior is substantially higher for VFD. Figure 1 shows an illustration to compare AFD with VFD.

### 2.3 Merits of AFD

AFD offers numerous benefits over VFD. Some articles highlight the benefits of AFD over VFD (Azizpour et al. 2025; Claussen, Ustad, et al. 2007). As the operation is carried out at atmospheric pressure, there is no cost involved in maintaining the vacuum inside the drying chamber. The initial investment cost is also lower than that of the VFD method. It operates at atmospheric pressure and, in principle, can be easily converted into a continuous operation, although this has yet to be reported in the literature. In contrast, attempts to make VFD continuous have led to semicontinuous batch designs due to the need to create a vacuum environment (Pyle and Eilenberg 1971). Additionally, the specific moisture extraction rate, which is the moisture extracted during drying per unit of energy consumed, of the AFD method is higher than that of the VFD method. AFD achieved a marked 1.1–4.2 kg/kWh higher specific moisture extraction rate than VFD (Bórquez et al. 2013). Though the quality of the product is preserved upon AFD drying, the samples dried by VFD outperform those dried by AFD. Nevertheless, the AFD sample shows reasonably good quality characteristics compared to VD and superior quality to other drying methods. Likewise, only slight differences in textural properties were observed compared to vacuum freeze‐dried eggplant cubes (Santacatalina et al. 2011). Besides these, the AFD method was found to be a feasible method for preserving bacteriophage with mannitol and trehalose. Owing to a lower temperature operation, AFD caused only 0.6 logs of titre loss, which is acceptable for the viability of the bacteriophage (Ly et al. 2019).

An interesting study related to the drying of itraconazole (frozen by thin‐film freezing) by AFD and VFD revealed that the powder produced by AFD has an electrostatically lower charge than the VFD‐dried powder. The powder dried by AFD or VFD exhibited a similar, amorphous nature and density. The reduced charge of the AFD‐dried powder was due to the larger size of the agglomerate and the larger pore size of the AFD powder, which resulted in a reduced surface area. Additionally, the AFD powder showed a higher residual solvent than VFD, which reduced the electrostatic charge. A lower charge is desirable for both industrial handling and improved drug delivery. Thus, the AFD‐dried powder was more desirable than VFD (O'Donnell et al. 2013). Such a study about the electric charge on the dried powder is vital as it determines the processability and application of the final product.

A cost saving of 30% can be accomplished by carrying out the freeze‐drying operation at atmospheric conditions (Donsì et al. 2001). Moreover, the AFD‐dried samples show slight differences in the quality, as observed by Carrion et al. (2018) for mushrooms dried by AFD (Carrión et al. 2018). AFD gives intermediate characteristics compared to VFD and hot air drying (Santacatalina et al. 2011).

### 2.4 Demerits of AFD

Though the AFD process is viable in terms of the cost economics of the process, the drying time in AFD is significantly higher than that in VFD. Vacuum drying offers a higher drying rate and better preservation of the product's quality characteristics. It took 5.5 h more in AFD to reach the same moisture content at the end of drying for strawberry slices (Rahman and Saidur 2016). Around 50% higher drying time was reported for carrots in AFD (20 h) as compared to VFD (10 h) (Boeh‐Ocansey 1984). Similarly, 80.77% more time was required to remove bound water using pulsed fluidized bed AFD as compared to the VFD method (Bubnovich et al. 2009). The reduced drying rates for AFD can be attributed to reduced internal diffusion. Under vacuum conditions, the temperature of the drying chamber can be kept higher in VFD than in AFD. The product's exposure temperature is near the ambient conditions in VFD. Additionally, the temperature of the food is further increased using a heating plate to desorb the moisture during the final drying stages. This higher temperature gives a vapor pressure gradient; it facilitates a higher moisture diffusivity in VFD than in AFD.

The provision to expose the product to these varied temperatures is possible in VFD, as the pressure is well below the triple point of water, which provides a broader range of temperature operation. On the flip side, as AFD operates at ambient pressure, even at a lower operating temperature, ice melting is possible, particularly if there is temperature ramping. The sample is in contact with cold, dry air moving at a certain velocity at atmospheric pressure; this ensures a higher external mass transfer coefficient. However, unlike the VFD process, the external environment is not an absolute vacuum. Moreover, as previously discussed, the operating temperature is also lower than the VFD process, resulting in a lower driving force due to a lower vapor pressure gradient.

The thermal conductivity of gases increases with an increase in pressure. When the sample is dried by VFD, the thermal conductivity of the atmosphere is poor, which makes the rate at which the heat is transferred to the sample the rate‐limiting step, whereas moisture diffusion is faster because of a high vapor pressure gradient, and internal diffusion is not the rate‐limiting step. In contrast, as AFD operates at atmospheric pressure, the thermal conductivity of the surrounding atmosphere is high. Hence, the rate at which the heat is transferred to the sample is high. However, the vapor pressure gradient is low, which limits the internal diffusion of moisture. At atmospheric pressure, the limitation of lower thermal conductivity of the sample is overcome; however, the effective diffusivity is reduced (Di Matteo et al. 2003). This results in an internal diffusion step as the rate‐limiting step for the AFD operation. This explains the lower drying rate of AFD.

Moreover, AFD can damage the sample's textural properties as opposed to VFD. This is again because of the previously stated reasons, related to the temperature of operation. The samples in AFD are more prone to melting and collapsing, deteriorating the textural properties. A shrinkage in carrot cuboids comparable to the convective heating methods was evident with AFD (Reyes, Vega, et al. 2010). Similarly, the surface area of the powder dried by AFD was less than that of VFD (Rogers et al. 2003). The VFD process is carried out in the presence of an absolute vacuum condition, which allows the ice to escape the food in the vapor phase without undergoing a liquid phase; this explains the better textural and structural quality of the VFD‐dried product. This is evident from some reported SEM analysis suggesting AFD‐dried samples had a higher shrinkage with a more compacted structure, unlike the VFD‐dried samples with a more porous structure. Nevertheless, a study conducted using fixed and temperature‐programmed AFD reported that garlic dried by the temperature‐programmed method produced a dried product of quality similar to that of a vacuum freeze‐dried sample (D. Xu et al. 2015)

A higher loss of color was also evident for carrots dried by AFD than for vacuum freeze‐dried samples (Boeh‐Ocansey 1984). A study conducted on the drying of apple slices using AFD reported a 39% higher degradation of polyphenols than the vacuum freeze‐dried sample and a 70% higher loss of ascorbic acid (Reyes et al. 2011). The AFD process is executed in the presence of a drying medium: air (low humidity). Oxygen in the air potentially expedites oxidative reactions, causing the degradation of antioxidants and the oxidation of pigments and bioactive compounds. These reactions are ruled out in the case of VFD. Another reason for greater damage to the product's nutritional profile is the prolonged process, which increases the exposure time of the bioactive to the oxygen‐rich environment.

### 2.5 Need for Intensification

The drying rate in AFD is typically relatively low in most of the reports in the literature. The AFD method's intensification can help reasonably reduce the drying time. The thermal conductivity of the medium is improved with the increase in the atmospheric pressure with AFD. However, the internal mass transfer is suppressed, which reduces the effective diffusivity. Despite a lower operational and initial investment cost, the increased time of operation is still a challenge. One can argue that reducing the pressure can enhance the rate of drying and help reduce the time; however, in a study carried out for freeze‐drying at varying pressure, it was demonstrated that even with the decrease in the pressure from 101.325 to 5.325 kPa, only a 1.16% decrease in the drying time was achieved (Liu et al. 2024). Additionally, as the name of the process suggests (AFD), decreasing the pressure defeats the simplicity of the process in undertaking sublimation at ambient pressure. Thus, this suggests that various strategies are required to accelerate the AFD process.

There are a few studies in the literature where the AFD method, with some assisted technology to accelerate the process, has helped to reduce the drying time to even shorter than the VFD method. For instance, mannitol, when spray‐dried in AFD conditions in a spouted bed drying, took only 3 h, which is much shorter than the VFD method (Men'shutina et al. 2005). Similarly, Bantle et al. (2009) compared the drying of a zooplankton species (*Calanus finmarchicus*) in fluidized AFD with that in VFD. The authors observed a higher drying rate of the AFD method than the VFD method.

**FIGURE 2:**
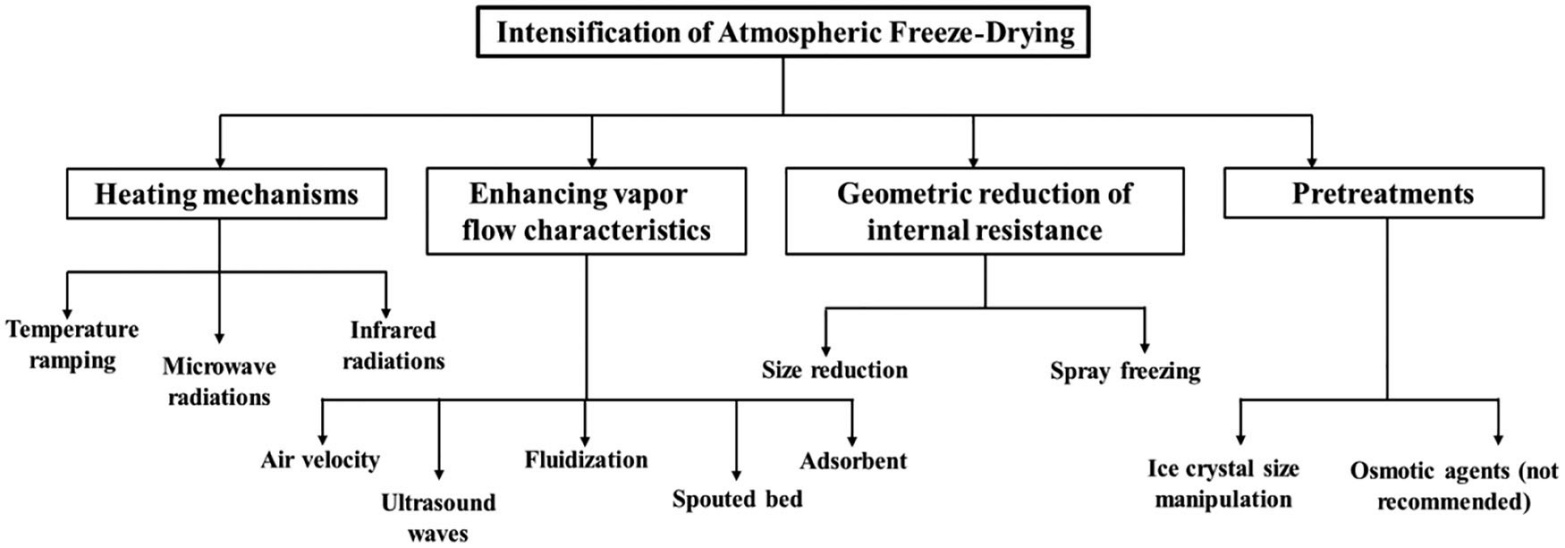
Classification for intensification of AFD.

When the intensification of the process was carried out using a temperature ramping program, garlic dried with AFD showed exactly similar quality characteristics to those of vacuum‐dried garlic (D. Xu et al. 2015). Thus, a proper strategy to intensify is required without compromising the quality of the dried food. The intensification can be brought about by employing different strategies; these strategies can be classified based on the heating mechanisms used, physical methods, reduction of internal resistance, and the use of certain pretreatments. Figure 2 gives a schematic of a proposed classification of the intensification method. Section 3 describes the different intensification methods investigated in the literature in greater detail.

## 3 Methods of Intensification

### 3.1 Intensification by the Heating Mechanism

#### 3.1.1 Temperature Ramping

Temperature plays an important role in freeze‐drying. The internal diffusion limiting step of AFD can be accelerated with the help of a temperature increment due to an increased vapor pressure gradient; this forms the basis of temperature ramping as a method of intensification of AFD. A 10°C temperature increment from −15°C to −5°C can lead to a 33% decrease in the drying time (Bubnovich et al. 2009). Similarly, a difference in the drying time of about 8 h was evident in reaching the same moisture content when the temperature was increased from −10°C to 5°C for the apple cube (10 mm) (Duan et al. 2013). It is quite common to observe that an increase in temperature is detrimental to the quality of the product. Despite this, studies suggest that a temperature ramping strategy adopted in such a way that it considers the inevitable changes in the physical properties of the food can help preserve the quality and reduce energy consumption, along with the reduction in drying time. The quality of the dried garlic slices was superior and comparable to the vacuum‐dried sample, and the energy consumption was also lower. Moreover, the time required to dry the sample was reduced from 44 h at −10°C to 26 h.

**FIGURE 3:**
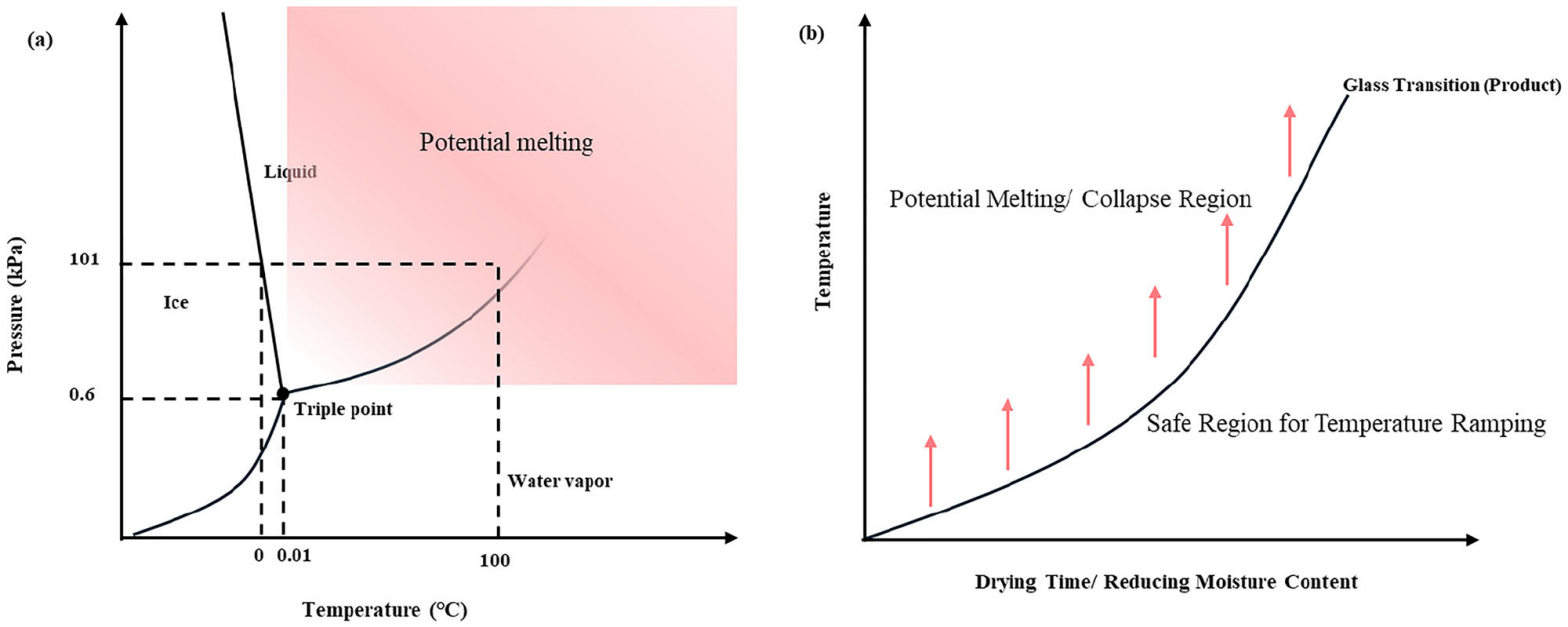
(a) Potential melting region for the food product during AFD. (b) Safe region for temperature ramping based on the glass transition temperature of the product during drying.

The physical properties of the product change dynamically with the change in temperature and with the drying process as the product is concentrated on removing moisture. When a temperature ramping approach is adopted for AFD, it should be carried out to maintain the solid state of ice during the drying operation. Besides, a very narrow range of temperature increments is available for AFD compared to VFD, as the conditions are above the triple point of water (Figure 3a). Even when the temperature is kept constant during the course of drying, implications still exist for the operating conditions. During the constant temperature drying process, the water present in the frozen state also undergoes thawing to some extent owing to the depression in the freezing point (D. Xu et al. 2015). The product's glass transition temperature increases as the drying process proceeds, and the degree of depression differs for different food products. A study carried out with an increased temperature for turnip, apple, and cod showed a different level of reduction in the drying time (Claussen, Strommen, et al. 2007). The difference in the drying time at −5°C and −10°C for cod fish and apple was 6.3 and 2.2 times, respectively. Increasing the temperature beyond the glass transition temperature causes the structural collapse of the food sample; this can severely affect the textural properties of the dried product. Figure 3b shows the safe margin that can be employed for temperature ramping during AFD. A significant change in the volume is evident if the temperature of the process is higher than the glass transition temperature of the material (Duan et al. 2013). An increase in the temperature from −10°C to 0°C caused a severe impact on the quality of the apple, affecting its porosity (Stawczyk et al. 2007). Thus, it is essential to understand the effect of temperature ramping on the product's quality characteristics while establishing intensifying strategies. Table 1 gives the different temperature ramping strategies adopted in the literature for different food products.

**TABLE 1:** Temperature ramping strategies used for AFD.

Sample	Method of drying	Temperature ramping	Findings	Reference
Carrot cuboids (6 × 6 × 1.5 and 6 × 6 × 3 mm)	AFD with pulsed fluidization	−5°C to 0°C or 5°C at 300 or 390 min from the initial time	Air temperature is the most important factor for moisture removal	Reyes, Vega, et al. 2010
Potato and carrot cubes (2 mm)	AFD with vibro‐fluidized bed	−10°C for 4 h, followed by −6°C for the rest of the drying period		Rahman and Mujumdar 2008b
Bovine intestine cubes (4 mm)	AFD with fluidized bed	At a low temperature for 5 h and then at an increased temperature for 2 h	Two‐stage temperature ramping can effectively improve the diffusivity and drying rate.	Senadeera et al. 2012
Murtilla fruit halves	AFD with fluidized bed and IR lamp	−5°C for 7 h, then 5°C or 15°C for 6 h	Temperature was the most effective factor in improving the drying rate	Reyes, Bubnovich, et al. 2010
Strawberry slices (*d* = 25 mm, *t* = 2 mm)	AFD	−10°C for 3 h and −5°C for 5 h	When the temperature was ramped, the process length was reduced by 20%	Rahman and Saidur 2016
Peas	AFD with fluidization	−5°C or −1°C for 9 h, then at 25°C		Alves‐Filho et al. 2004
Spherical particles of commercial cereal food paste (*d* = 2 mm)	AFD with adsorbent (zeolite)	−10°C to 40°C		Lombraña and Villarán 1997
Apple cubes (10 mm)	AFD	−5°C for 0–6 h, −10°C for 6–20 h, 5°C for 20–24 h, 20°C for 24–28 h, and 40°C for 28–34 h	Ramping of temperature reduced the drying time to 34 h, which was 64 and 56 h at −10°C and −5°C. Ramping of temperature also helped with the preservation of the quality characteristics of the product, similar to the samples dried at −10°C.	Duan et al. 2013
Ertapenem (an antibiotic) and trehalose (a biologic stabilizer)	Atmospheric spray freeze‐drying	−20°C for first 20 min, −20°C for 2 h, 15°C for 1 h, −5°C for 1 h, 5°C for 1 h, 25°C for 1 h	Drying completed in 7 h	Ly 2019
Garlic slice (5 mm)	AFD	−5°C for 8 h, −10°C for 8–12 h, −15°C for 12–20 h, 40°C for 20–26 h	Temperature ramping helped in getting a good‐quality product with lower energy consumption.	Duan et al. 2015

From Table 1, it can be clearly understood that most of the work in the literature on AFD with temperature ramping relies on two‐stage temperature ramping. However, it is important to note that there is a lack of understanding of the logic behind the increase in temperature and at what stage it should be adopted. Only a few studies focus on determining the strategies to ramp up the temperature. In 1984, Ocansey carried out the drying operation of AFD with a systematic temperature ramping program; the method resulted in a reduction of drying time from 20 h for the carrot at −10°C to 11 h with temperature ramping (Boeh‐Ocansey 1984). The temperature was increased when the drying rate was found to decrease. D. Xu et al. (2015) programmed temperature ramping based on the depression in the freezing point of the sample throughout the drying process. Similarly, Duan et al. (2013) used the glass transition temperature and the comparison of drying rate at the beginning of the process to ramp the temperature and successfully reduced the drying time to 34 h from 56 and 64 h at −5°C and −10°C, respectively (Duan et al. 2013). Among these different strategies, the ramping based on the glass transition seems to be most appropriate for AFD, as the temperature is the most limiting parameter for the operation, as discussed previously. The increment based on the receding drying rate or depression in the freezing point poses a risk of a rise in the temperature to a level above the glass transition temperature of the sample, leading to immediate melting of the sample. However, any of the mentioned strategies require continuous monitoring of moisture content and determination of the property, for instance, the glass transition temperature at a given moisture content, and deployment of temperature ramping lower than the glass transition temperature. However, monitoring the moisture content in an industrial setting where tonnes of produce are processed is not feasible. Most industrial‐scale operations rely on the temperature of the feed to determine the endpoint. This put forth a gap between lab‐ and industrial‐scale operations, where inefficiencies in determining the moisture can potentially be a challenge when deploying temperature ramping strategies.

#### 3.1.2 MW

**FIGURE 4:**
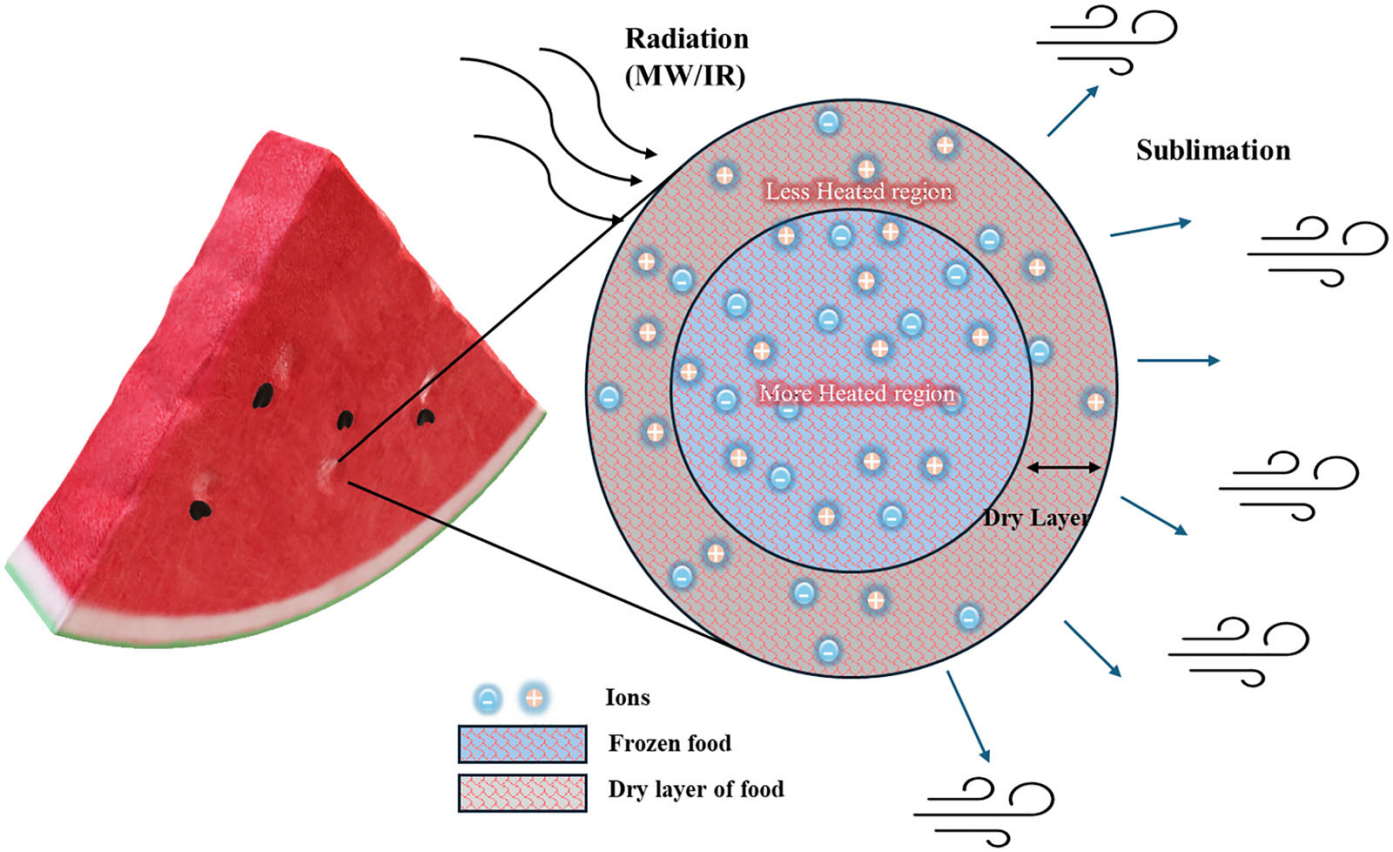
Acceleration of AFD using radiation (MW/IR). Watermelon image adapted from Microsoft PowerPoint.

The principle behind MW heating of food largely depends on the food's dielectric properties. MWs interact with the food's polar (mainly water) molecules, causing a continuous rotation of the dipolar water molecules, generating heat. However, in the frozen state, the kinetic and intermolecular energies, along with the distribution of the dipolar molecules of water, are found to be unchanged in the application of MW to pure ice within a frequency range of 2.5–20 GHz (Tanaka and Sato 2007). This is because of the water molecules’ rigidity due to strong hydrogen bonds. For food applications, 2450 MHz is the most used. The ice still showed a very small heating rate with the application of MW. The dielectric constant of the ice is 3.2 (−12°C), whereas, for water, it is 78.2 (20°C) at 2450 MHz. Food is a complex mixture, and along with the moisture, it also shows the presence of ionic salts and polar molecules, which also contribute to the dielectric properties of the sample. The sample's dielectric constant and loss factor determine the food's interaction with the applied MW radiation. This interaction of MW with the polar molecules in food alleviates food temperature, which increases the vapor pressure gradient and improves the drying rate. Figure 4 shows the mechanism involved in the acceleration of AFD using radiation. Food's dielectric constant and dielectric loss factor increase with temperature (Klinbun and Rattanadecho 2017). The temperature of the food increases as the AFD process proceeds (Nakagawa et al. 2020).

MW‐assisted AFD has shown quite promising results in terms of the drying rate achieved. A simultaneous application of MW during AFD of peas reduced the drying time by 50% at 280 W (Eikevik et al. 2012). The quality of the food sample can also be preserved using the MW‐assisted AFD method.

MW‐assisted fluidized bed drying with a pulsed vacuum of edible fungus reduced the drying time by 71.9% (Wu et al. 2019). Furthermore, the increase in the MW power reduced the drying time. However, the improved rate was achieved at the cost of the product's quality deterioration. The sample's color and porosity were severely affected at higher power. The thickness of the food is also important when applying MWs for intensification during AFD. The penetration depth of MW reduces with an increase in temperature for a given thickness (Klinbun and Rattanadecho 2017). This implies that the effectiveness of MWs will be reduced in the later stages of drying. However, no such report has been made about the inefficiency of MW in a later stage of drying. One possible reason for this could be the thickness of the sample; for thin slices, the penetration depth is not the limiting factor. However, this penetration depth becomes relevant for samples with larger thicknesses.

#### 3.1.3 IR Radiation

Similar to MW radiation, the effect of IR in assisting AFD has also been investigated by a few studies. However, the results were not as promising as those of MWs. The interaction of IR radiation is similar to that of MW radiation with the polar molecules in the food sample. However, the penetration depth of IR is lower than that of MW. Thus, the application of IR is not as effective as MW. The intensification of AFD with the IR‐assisted method is found to be effective for thin samples. For instance, with the implementation of IR during AFD, a 60% reduction in the time required for the primary stage of drying carrot slices (0.75–3 mm) was evident (Bubnovich et al. 2009). Another study reported a significant effect of IR radiation on apple slices (4‐ and 8‐mm thick) (Reyes et al. 2011). Similarly, the application of radiation along with temperature ramping helped improve the drying rate for carrot and potato cylinders (5‐ to 16‐mm thick) (Rahman and Mujumdar 2008a). However, some studies reported IR to be ineffective in improving drying rate, specifically for murtilla berry halves (average size of berries: 10–20 mm). The higher thickness of the sample can be attributed to the inefficiency of the IR. The limited penetration depth of IR can be one of the reasons for the insignificant effect of this method compared to MW. One study also observed that only 10% of the power from IR was effectively received by the samples (Reyes et al. 2011). Furthermore, the same study observed that the effect of IR was significant in the earlier drying stages and diminished during the later stages. The phenomenon related to the decrease in the penetration depth of the radiation with the increase in the product temperature during the later stages of drying can explain the observed ineffectiveness of this method.

Limited work on IR as an intensification method for AFD isavailable. Table 2 gives the radition based methods employed for intensification of AFD. Studies on the effect of thickness and drying time on the diminishing effect of IR, and a comparison of IR and MW for the intensification of AFD, are required. This is particularly important for AFD because the constraint of maintaining the temperature of the sample below the glass transition temperature is always there, and with MW, a higher absorption of radiation can increase the product temperature; this rise in temperature could be anticipated to be lower with IR, reducing the risk of product collapse.

**TABLE 2:** Microwave and infrared methods employed for intensification of AFD.

Intensification method	Sample and freezing method	Conditions	Findings	Reference
Microwave	Pea (*d* = 8.45 mm), frozen at −30°C	−6°C, 4.5 m/s, RH: 22.5%, 0 W, 140 W, and 280 W	Drying time was approximately halved at 280 W	Eikevik et al. 2012
	*Cordyceps militaris* (Edible fungus), frozen at −40°C	−40°C, 660 W, 760 W, and 860 W, fluidization only for 0.1 s every 10 min	A 71.9% reduction in drying time by MW. The rehydration time was faster than that of the hot air drying. Samples comparable to VFD.	Wu et al. 2019
Infrared	Murtilla fruit halves, frozen at −18°C or immersed in liquid nitrogen for 5 min	−5°C for 7 h, then at 5°C or 15°C for 6 h. A 2‐m/s airflow and an IR lamp were placed at 0.3 m.	Due to the lower efficiency of the system, IR was not effective in improving the drying rates.	Reyes, Bubnovich, et al. 2010
	Apple slices (40 × 12 mm and 4‐ and 8‐mm thick), frozen at −18°C or by immersion in liquid nitrogen for 15 min.	−5°C for 10 h, then 15°C or 25°C, 2 m/s	Only 10% of the effective lamp power was received by the sample. IR has a significant effect on the drying kinetics. The effect of IR diminishes over time.	
	Carrot slices (0.75, 1.5, and 3 mm), frozen at −20°C	−5°C, 2 m/s	60% reduction in the primary drying time	Bubnovich et al. 2009

### 3.2 Intensification by Enhancing Vapor Flow Characteristics

#### 3.2.1 Air Velocity

The air flow plays the most important role as the drying medium. The properties of the drying medium, such as the temperature, relative humidity, and the velocity of the air, are important parameters affecting the drying rate. Temperature as a parameter has been previously discussed. The relative humidity of the air in AFD is maintained as low as possible to ensure a higher vapor pressure gradient. However, it is difficult to control the relative humidity; hence, the study based on the effect of relative humidity of the air on the drying kinetics is absent. The third important parameter, which is the velocity at which the air is passed through the drying chamber, affects the drying kinetics to some extent. The freeze‐drying process involves the diffusion of vapor from the ice front to the surface of the food through the dry layer due to the vapor pressure gradient. Once the moisture reaches the surface, it diffuses into the cold, dry air in the vicinity; this external mass transfer is convective for AFD. The velocity also increases the external heat and mass transfer coefficient. A formal analysis of the effect of velocity on the external heat and mass transfer coefficient in AFD is not available. However, the impact of velocity on the internal diffusion of moisture within the product was found to be insignificant as reported by Santacatalina for apple cubes measuring 8.8–17.5 mm (Santacatalina et al. 2015). On the contrary, a study conducted on beef meat (1‐ to 3‐mm thick) found that applying the impinging jets on the food sample improves the drying rate compared to the cross‐flow configuration (Y. Xu et al. 2024). Applying the impinging jets reduced the thickness of the boundary layer during AFD; the jets improved the mass flux rates by 38%–51% (compared to cross flux) during the initial drying stages for thin samples. These studies suggest that the effect of velocity on the drying rate is significant when thin samples are dried. This is because the internal diffusion is the rate‐limiting step in AFD; the velocity has no significant effect on the internal diffusion of moisture when the length of the internal diffusional path is high.

#### 3.2.2 US

**FIGURE 5:**
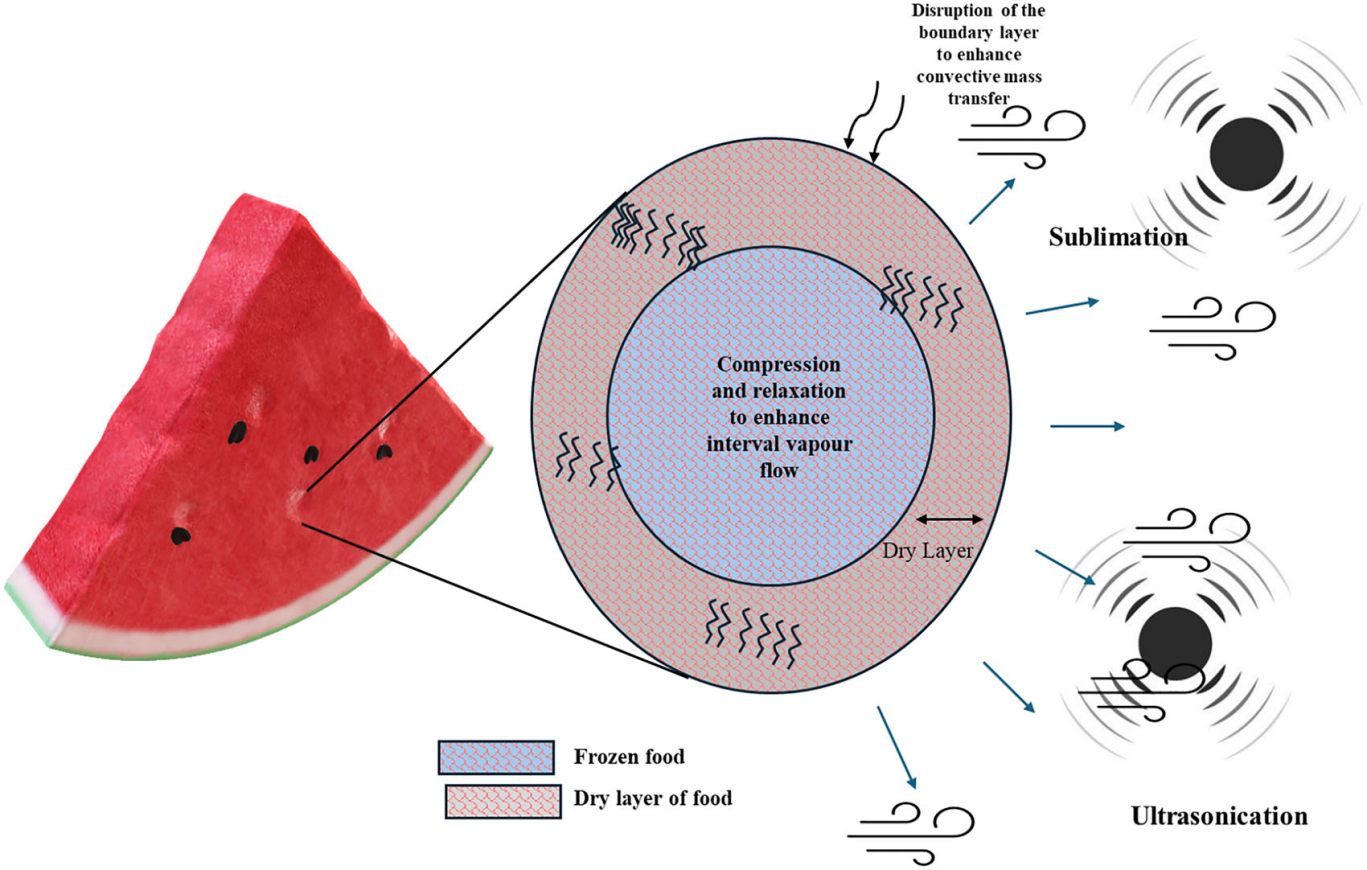
Ultrasound‐based acceleration of AFD. Watermelon image adapted from Microsoft PowerPoint.

Internal diffusion is the rate‐determining step in AFD. US is an effective method to increase the internal diffusion during drying operations. Applying US to the AFD process enhances the mass transfer rate. US induces a rapid compression and expansion of the US wave, which causes the creation of alternative channels that facilitate the movement of water vapor (Santacatalina et al. 2015), and it also disrupts the external boundary layer. Figure 5 shows the mechanism involved in US‐based acceleration of AFD. The improved mass transfer rate explains the increase in the effective diffusivity with US. The mass transfer rate was found to increase by 23.2% (Bantle et al. 2010). Additionally, the attenuation of US acoustic waves also provides the energy required for the sublimation of water. US waves attenuate in the presence of a medium, which certainly increases the temperature of the product and its vicinity. This phenomenon is absent in US‐assisted VFD because of the vacuum. The increase in the temperature of the medium or sample during US AFD has not been investigated yet, but a study with US‐assisted hot air drying (40°C and 60°C) at 2 m/s for pork liver revealed a 2.4°C–2.5°C increase in the air temperature (Sánchez‐Torres et al. 2025). Several studies have been reported in the literature investigating the effect of US on AFD. Undoubtedly, US can potentially increase the drying rate by increasing the effective internal diffusivity. However, the increase in the internal diffusivity was surprisingly varied for different products, as reported by other studies. Bantle and Eikevik (2011b) observed that the increase in diffusivity can be as high as 14.8% for peas. Meanwhile, a 380% increase in the effective diffusivity was seen for eggplants with 25‐W US (Colucci et al. 2017). With the increase in the power of the US transducer, the effective diffusivity was found to increase. The effective diffusivity was increased by 38% and 24.7% for ice particles and peas (Bantle 2011). A 123.5% increase in the moisture diffusivity with the application of US was observed (Santacatalina et al. 2016). The differences in the increase in the effective diffusivity can be attributed to the varied nature of the sample and the US operating parameters. Consequently, the increased internal diffusivity reduces the drying time. A 58.5% and 78.2% reduction in drying time was reported for button mushroom slices dried in AFD for 12.3 and 24.6 kW/m^3^ US powder density, respectively (Carrión et al. 2018). A 60% reduction in drying time, compared with AFD, was reported when US at 20.5 kW/m^3^ was applied to carrot cubes (Santacatalina et al. 2012). Similarly, a 35%–54% shorter drying time was seen for cod fish (Ozuna et al. 2014).

The increase in the diffusivity is also dependent on the applied power of the ultrasonication. At a power of 15.2 and 68.2 W, the effective diffusivity increased from 7.6% to 13.7%, respectively (Bantle and Eikevik 2011b). Likewise, a 159.2% and 280.6% increase in the effective diffusivity was observed for mushroom slices at 12.3 and 24.6 kW/m^3^, respectively (Carrión et al. 2018). This is because the mass transfer coefficient increases up to fourfold when the power of US is increased (Colucci et al. 2017).

Another important aspect of US is that it effectively improves the diffusivity during the initial hours of drying, and the effect is insignificant in later stages (Bantle and Eikevik 2011b). Likewise, the temperature also influences the effectiveness of US, as the effect of US reduces with an increase in the temperature (Mello et al. 2020; Bantle 2011). These peculiar observations regarding the effectiveness can be attributed to the mechanisms by which US operates. The important phenomenon occurring because of the cavitation is related to the interference of US with the boundary layer and the attenuated heating.

US accelerates the drying rate at the air–product interface as the drying proceeds, and the thickness of the dry layer increases, which explains the reduction in the effectiveness of US during the later stages of drying. In the context of temperature, as the temperature increases, the effective diffusion of moisture increases owing to the rise in temperature, and hence, the application of US does not significantly affect the diffusion of moisture at high temperatures. Moreover, denser samples absorb more ultrasonic waves than porous samples (Merone et al. 2020). The density of the ice slightly increases as the temperature decreases (below the subzero conditions). As the density increases at lower temperatures, food samples absorb more US at low temperatures, which enhances the effectiveness of the cavitation phenomenon (Merone et al. 2020). Thus, the US effect is more predominant at lower temperatures.

The geometry and size of the product also play a vital role in determining the rate of drying by US‐assisted AFD. Slab geometry experienced an apparent increase in the drying rate by 92%, compared with cylindrical geometry, where the drying rate increased by 88% (Moreno et al. 2017). The study also suggested that US is more effective in accelerating the drying process of larger particles than smaller ones. Another study also confirms this (Colucci et al. 2017). This is because larger samples provide a lower surface‐to‐mass ratio, which increases the absorption and penetration of US compared to a small sample (with a high surface‐to‐mass ratio).

Though the method helps enhance the drying rate, one of the major drawbacks is the inevitable loss of bioactives and deterioration of the quality of the dried product. It is observed in several studies that ultrasonication causes the production of free radicals and can reduce antioxidant activity. Colucci et al. (2018) observed that the application of US to eggplant during AFD increased the antioxidant degradation from 48.1% to 58.7% at 25 W (effect not significant, only a slight reduction) (Colucci et al. 2018). A significant increase in the total color change and browning of the button mushroom slices was also reported (Carrión et al. 2018). The effect of the color change and browning of the sample could be attributed to the reactions due to an increase in the temperature of the sample. The rehydration time was also observed to be higher in the same study due to structural damage. The structural damage is found to be insignificant at lower US power and increases with the applied power of US. The authors also reported an increase in the product temperature in US applications during AFD. A larger sample experiences a greater rise in temperature compared to a smaller one (Colucci et al. 2018).

Considering the energy aspect of the process, US can consume up to 50% of the relative energy among other components of US‐assisted AFD. Nevertheless, a 70% reduction in the overall energy consumption can be achieved as the drying time is reduced (Merone et al. 2020). Table 3 summarizes the different physical methods applied for the intensification of AFD.

**TABLE 3:** US‐ and fluidization‐assisted mode for intensification of AFD.

Intensification method	Sample and freezing method	Conditions	Findings and reference	Respectively
US	Apple cube (8.8 or 17.5 mm) and cylinder (*d* = 15 mm, *h* = 40 mm), frozen at −18°C.	5°C, 10°C, and 15°C at 2, 4, and 6 m/s with 25‐, 50‐, and 75‐W US	370% increase in the effective diffusivity of the sample at 25 W	Santacatalina et al. 2015
	Eggplant cubes (8.8 and 17.6 mm)	−5, −7.5, and −10°C, with 0‐, 25‐, and 50‐W US at 21.9 kHz and 2 and 5 m/s	Degradation of antioxidant activity by 58.7% and 53.2% at 25‐ and 50‐W US. A 66.2% and 66% reduction in total phenolic content at 25 and 50 W, respectively.	Colucci et al. 2018
	Button mushroom slices (6 mm), frozen at −32°C	−10°C, 2 m/s at 24.6 and 12.3 kW/m^3^ (21.9 kHz)	US significantly reduced the time for drying. A 58.5% and 78.2% reduction in the drying time was observed at 12.3 and 24.6 kW/m^3^ of US power density. A 159.2% and 280.6% increase in the effective diffusivity of the moisture was evident at 12.3 and 24.6 kW/m^3^, respectively. US significantly affected the color change. Higher power US led to more structural damage.	Carrión et al. 2018
	Carrot cubes (10 mm), frozen at −18°C.	−10°C, 10% RH, 20.5 kW/m^3^	A 73% higher diffusivity by US application. A 60% reduction in the drying time. Higher rehydration capacity of US‐dried carrots.	Santacatalina et al. 2012
	Eggplant cubes (8 and 17.6 mm)	−10°C and −5°C; 2 and 5 m/s; 0, 25, and 50 W; 20.5 kW/m^3^	A 72% and 82% time reduction at 25 and 50 W, respectively. At high power (50 W), the effective diffusivity is one order of magnitude higher compared to without US. Mass transfer coefficient increases by up to fourfold with US.	Colucci et al. 2017
	Desalted cod fish parallelopiped (50×30×5 mm), frozen at −18°C.	−10°C, 2 m/s, 20.5 kW/m^3^, RH below 10%	A 16% and 60% reduction in the drying time at 0°C and −10°C, respectively. A 123.5% increase in the moisture diffusivity at −10°C. Faster rehydration and more water‐holding capacity.	Santacatalina et al. 2012
	Salted cod fish parallelopiped (50×30×5 mm), frozen at −18°C	−10°C, 0°C, 10°C, and 20°C, 2 m/s, 9% RH, 20.5 kW/m^3^	A 35%–50% reduction in the drying time. A 42%–110% improvement in the effective moisture diffusivity. A higher rehydration ratio.	Ozuna et al. 2014
Fluidized bed	Pea (*d* = 8.45 mm), frozen at −30°C.	−6°C, 4 m/s, 22.5% RH	Fixed bed drying was better than fluidized bed drying. Diffusion was higher in a fixed bed compared to a fluidized bed.	Eikevik et al. 2012
	Pea, frozen at −20°C	−5°C, 4 m/s	The drying rate was minimum in fluidization compared to MW‐assisted and hot air drying. Fluidization, along with MW, was beneficial for maintaining the quality of the product.	Zielinska et al. 2013
	Fresh shrimps (4, 6, and 8 mm), frozen at −20°C and −10°C, and with liquid nitrogen	−5 and −15°C, with zeolite and bran as adsorbents; 18, 20, and 26 cm/s	Fluidization helps to avoid the saturation of the adsorbent used. Fluidization provides a higher drying rate in the primary drying stage.	Donsì et al. 2001
	Biological product (peeled and reduced to small pieces), frozen at −18°C.	−1°C, −4°C, and −6°C at 3, 4, and 5 m/s	An increase in the fluidization velocity, keeping other parameters constant, reduced the drying period.	Tomová et al. 2004
Vibrofluidized	Potato and carrot cubes (2 mm), frozen at −22°C.	−10°C and −6°C. Air flow rate at 0.0188 m^3^/s	A higher rate of drying with the vibrating bed. Absence of a falling rate period with vibration.	Rahman and Mujumdar 2008b

#### 3.2.3 Fluidization

The fluidization of the food material to accelerate the AFD process is one of the most used methods in the literature. The rate of drying can be significantly increased by applying fluidization (Donsì et al. 2001). The fluidization increases the heat and mass transfer coefficient enormously. A 20–40 times increase in the heat transfer coefficient as compared to VFD was reported (Boeh‐Ocansey 1984). Constant powder fluidization of danazol powder during drying in AFD caused a markedly higher rate than the vacuum‐freeze dried powder (Rogers et al. 2003).

A decrease in the drying time was observed by Tomova et al., with an increase in the flow rate of the drying medium during fluidization as the external mass transfer coefficient increases (Tomová et al. 2004). However, the effect on the diffusion depends on other factors such as the construction of the drying chamber, fluidization velocity, material characteristics, and so on. One of the studies observed that the diffusion was higher for a fixed bed AFD of peas rather than the fluidized state (Eikevik et al. 2012). The lower diffusion rate was attributed to a faulty construction or the bypass effect. Also, it is worth noting that the application of fluidization to food can simply subject it to mechanical damage and, in turn, hamper the quality of the product.

Few studies have also conjugated fluidization with other methods of intensification of AFD, such as MW and US. Surprisingly, the application of fluidization failed to show the expected results. Fluidization potentially reduces the effect of ultrasonication, as observed by Bantle and Eikevik (2011a). The diffusion of moisture was decreased from 24.7% in the fixed bed (1 m/s) to 8.5% in the fluidized state. Effective diffusivity was found to be reduced with increased applied velocity. The increased velocity reduces the contact time between suspended peas and the air. Moreover, it also interferes with the interaction between US and the sample. Likewise, fluidization (4.5 m/s) was not as effective as the fixed bed (2.7 m/s) MW‐assisted AFD (Eikevik et al. 2012; Zielinska et al. 2013). Nevertheless, fluidization can be made effective by using different strategies; a few studies showcase this by applying fluidization in pulsed mode or using the vibro fluidization technique. The purpose of using pulsed or vibrational motions in fluidization is to improve heat transfer, maintain a uniform temperature distribution, and increase drying efficiency. A 71.9% reduction in the drying time was achieved with a pulsation of 0.1 s for 10 min during MW‐assisted AFD (Wu et al. 2019). Rahaman and Mujumdar first introduced vibro fluidization to AFD, which further increases the drying rate. Furthermore, the effect of vibration is more significant during the initial stages of drying. A characteristic constant rate of drying was evident with vibro fluidization, contrary to a falling rate period without vibrations (Rahman and Mujumdar 2008b). This suggests that vibrofluidization helps enhance the external mass transfer rate; as the sample gets dried, the thickness of the dry layer increases, and the ice front moves to the interior of the food sample. Thus, the vibrofluidization is ineffective in the later stages.

Spouted bed drying is known to overcome the shortcomings of fluidized bed drying, such as agglomeration, channelling, product deformation, and so forth. However, not much work has been done on spouted bed AFD, and a comparison of spouted bed AFD with fluidized bed AFD has not been made. The drying was quick when the spouted flow was applied to the mannitol solution. Coupled with SFD, the spouted bed dryer helped to complete the drying process of 15% aqueous mannitol solution in just 3 h, substantially shorter relative to the conventional VFD (Men'shutina et al. 2005).

#### 3.2.4 Use of Adsorbent

The main problem with AFD is that the vapor pressure gradient is low at low‐temperature operation. The air passed at a low temperature possesses a lower capacity to hold moisture, which eventually leads to a lower drying rate. To overcome this effect, recent studies have shown that using adsorbent material in drying operations has become quite promising. The adsorbent helps in two ways: the moisture from the sample readily gets absorbed into the adsorbent because the heat of sorption of the moisture and the sublimation heat are of the same order of magnitude; therefore, the heat released during the sorption process (exothermic) can be used for the sublimation of ice to vapor (endothermic), and the drying agent, that is, the air in the vicinity of the sample, is not saturated (preventing localized saturation) with moisture, thereby maintaining the vapor pressure gradient for the drying operation (Coletto et al. 2017). A reduction in the drying time from 8 to 6 h was evident with the use of gel particles as adsorbent at a 1:1 adsorbent‐to‐sample ratio for drying potatoes and carrots (Rahman and Mujumdar 2008b).

The factors affecting adsorbent‐assisted freeze‐drying include the type of adsorbent used, the sample‐to‐adsorbent ratio, the fluidization velocity, and the chamber temperature. An increase in the fluidization speed helps prevent the saturation of the adsorbent and also reduces the time of the primary stage drying (Donsì et al. 2001). The most used adsorbents are activated carbon, activated alumina, silica gels, and starch particles. It was observed that the zeolite, as an adsorbent, outperformed bran for fluidized AFD of shrimps (Donsì et al. 2001). Similarly, Di Matteo et al. (2003) also showed that zeolite exhibited the highest drying rate compared to other adsorbents used. They also reported that the corn starch formed agglomerates during the drying process, leading to mechanical damage to the product. Table 4 presents the different adsorbents used for AFD food drying and their effects on the drying process.

**TABLE 4:** Effect of using adsorbent for AFD food drying.

Sample	Adsorbent	Conditions	Effect of using an adsorbent	Reference
Potato and carrot cubes (2 mm)	Gel particles (*d* = 3 mm) adsorbent to product ratio = 1:1	−10°C and −6°C, with adsorbent to product ratio as 1:1, 0.0188 m^3^/s of flow rate, with vibration	Drying time was reduced from 8 to 6 h.	Rahman and Mujumdar 2008b
Shrimp	Bran and zeolite	−5°C and −15°C, with zeolite and bran as adsorbents; 18, 20, and 26 cm/s	The adsorbent helped improve the drying rate. Zeolite showed a higher adsorption capacity than bran. The rehydration ratio of the sample dried with bran was higher.	Donsì et al. 2001
Potato cylinder (*d* = 6, 8, and 11 mm; *h* = 10 mm)	Starch, bentonite, bran, zeolite, maize flour	−10°C and −5°C	Zeolite showed the highest drying rate. Cornstarch caused mechanical damage to the sample. Starch formed an agglomerate during fluidization and stuck to the product.	Di Matteo et al. 2003
Potato parallelopipeds (3 mm × 1 cm), *t* = 2, 3, and 5 mm.	Pregelatinized corn starch (*d* = 160 µm)	−65°C; 300 g of sample immersed in 5 kg of adsorbent.	Starch particles stick to the product, making it difficult to separate after drying.	Wolff and Gibert 1990a

However, certain issues exist with the use of adsorbents for drying food and pharmaceutical ingredients. The problem is related to the compatibility of the adsorbent with the food material. Most of the adsorbents used are synthetic in nature, and during the drying process, there is direct contact of these materials with the food, rendering it unfit for human consumption. Separating the adsorbent from the food material is also a difficult process, posing another disadvantage of their use (Bubnovich et al. 2009; Marques et al. 2007). In a study conducted for drying potatoes with corn starch as the adsorbent, it was observed that the adsorbent particles tended to stick to the food sample, making it difficult to separate (Wolff and Gibert 1990a). Once the freeze‐drying is completed, the adsorbent needs to be regenerated. The regeneration process adds to the processing cost and depends on the regeneration temperature. A regeneration temperature higher than 70°C is ineffective, as observed by Wolff and Gibert (1990b) for starch as an adsorbent.

### 3.3 Intensification by Reducing Internal Resistance

#### 3.3.1 Size Reduction

The size of the sample holds paramount importance when reducing the drying time. The rate of drying varies inversely with the size of the sample. Garcia‐Perez et al. observed a dominating falling rate period with a continuous reduction in the drying rate when a pressed cod fish slab was dried in a fluidized bed atmospheric dryer (Garcia‐Perez et al. 2005).

When the thickness of the slice was reduced to half, a 76% reduction in the time for moisture removal in the primary drying stage was observed for carrots (Bubnovich et al. 2009). Moreover, Donsì et al. (2001) observed a fourfold increase in the drying time by doubling the thickness of the sample (shrimp). Additionally, it is important to understand that, as the internal diffusion of moisture is the major bottleneck in removing moisture from the food for the AFD process, the reduction in size is more effective in reducing the drying time than the VFD process. This is because in AFD, when the size is reduced, the effective internal diffusional path for moisture is reduced, which is the rate‐limiting step for AFD. On the other hand, in VFD, the external mass transfer is rate‐limiting; hence, reduction in the size is not as effective as AFD to increase the drying rate. Drying potatoes with a thickness of 5 mm to reduce the moisture content to 50% of the initial moisture took 6 and 2 h in AFD and VFD, respectively. In contrast, when the thickness was reduced to 3 mm, there was a marked reduction in drying time to 2 h for AFD and a nominal reduction in the case of VFD to 1.5 h (Wolff and Gibert 1990a). The study emphasizes that the reduction of particle size for AFD is more effective in reducing the drying time than for VFD. Similarly, it was observed that below a certain critical thickness of the carrot slice, the drying rate was higher for AFD than VFD. However, this trend was flipped with an increase in the thickness of the carrot slice (Boeh‐Ocansey 1985). This suggests that the AFD process is more sensitive to an increase in the thickness, negatively affecting the drying rate. When the pressure is high, heat transfer is faster than under absolute vacuum conditions. However, when absolute vacuum conditions exist, the rate of moisture transfer from within the sample to the surrounding area is faster because of a higher concentration gradient, which explains the enhanced drying rate of AFD for thin samples.

While the thickness of the sample influences the drying rate, its shape and geometry are equally important. For a given dimension, a sample with a different geometry, such as a plate, cylinder, or sphere, can have a different drying time (Wolff and Gibert 1990b). Similarly, another study also observed differences in drying time among different geometries. A slab of apple experienced a lower drying rate compared to the cylinders, with a 37% higher drying time compared to the apple cut into cylinders (Moreno et al. 2017). Apart from these conventional studies on size reduction, Bantle applied some modifications to the process; the food sample was crushed after the freezing step, which essentially reduced the particle size of the food. The resulting food sample showed a marked increase in the drying rate of AFD compared to VFD. The increase in the rate of drying can be explained by the reduced internal resistance offered by the smaller particle size. The drying time of CF was 24 h in AFD, whereas it took 48–60 h to reach the same moisture content in VFD (Bantle 2011). Moreover, the losses in the phospholipids and the composition of free fatty acids in the food sample during drying were comparable to VFD.

#### 3.3.2 SFD

The method was introduced in the 1990s, especially for the heat‐labile constituent. SFD involves spraying a solution into a cold medium and freeze‐drying the resultant frozen particles, which can be performed by contacting the particles with a cold, dry gas stream in a fluidized bed dryer, typically at atmospheric pressure. The SFD can dramatically improve the drying rate of the slurry compared to the conventional VFD operation. This is because the atomization of the feed causes the size reduction, which helps in the intensification of AFD. In the context of AFD, once the atomization and freezing of the sample are concluded, the drying proceeds in a low‐temperature condition at atmospheric pressure. The method is most applied for freezing liquid extract along with different wall materials or protectants. The flow rate of the feed and the concentration of the feed are critical aspects determining the rate of drying in this method. A higher flow rate of the feed improves the drying rate. Similarly, a higher concentration of the solids in the feed also positively impacts the drying rates (Mumenthaler and Leuenberger 1991). The drying process can be completed in a few hours, as opposed to several hours to days in the conventional freeze‐drying process. AFD of the antibiotic ertapenem, along with trehalose as a stabilizer, was completed in 7 h with the help of temperature ramping and SFD operation, compared with a few days required in conventional VFD operation (Ly 2019). The drying of skim milk or mannitol along with prebiotics was completed within a few hours using the SFD method (Wang et al. 2006). It is important to understand that most studies reported in the literature comparing atmospheric and vacuum SFD compare the drying rate of spray‐frozen powder in AFD with that of the formulation dried in VFD. An appropriate way of comparison would be to spray‐freeze the formulation, followed by freeze‐drying using AFD or VFD. Nevertheless, Rogers et al. have made such comparisons for a drug formulation. The drug danazol and other excipients were dissolved in tetrahydrofuran as a solvent and were sprayed frozen into the liquid to get the frozen powder. The frozen powder was then dried in both AFD and VFD. It was observed that 12 h at −30°C was sufficient to remove the solvent by AFD, whereas 24 h was required in VFD at −40°C. Similarly, the secondary drying phase to remove the bound water in VFD was 34 h long at 25°C, which only took 12 h in AFD (Rogers et al. 2003).

The limitations of atmospheric SFD are commonly associated with the enormous quantities of air as a drying medium required for this method, which increases the cost of production (Anandharamakrishnan et al. 2010). Besides this, the method is suitable only for slurry‐like or liquid samples.

### 3.4 Intensification by Pretreatments

#### 3.4.1 Ice Crystal Size Manipulation

The ice crystals play a crucial role in determining the rate of drying. It is well‐known that the slow‐freezing method yields a higher drying rate in VFD than the quick‐freezing method. The drying rate is observed to be high when the size of the ice crystals is large (slow freezing method) (Marques et al. 2007). The theory also holds for AFD. Few studies have explored the effect of freezing methods on the drying rates of AFD. Reyes, Vega, et al. (2010) observed a higher drying rate for carrots frozen in the freezer at −18°C than those frozen using liquid nitrogen. The moisture ratio of the carrots after 400 min of drying was approximately 0.45 and 0.35 for quick freezing and slow freezing, respectively. Similarly, the smaller size of the ice crystals generated by the quick freezing method led to a lower drying rate for apple slices as compared to the slices with larger ice crystals (Reyes et al. 2011). After 10 h of drying, the moisture ratios for quick and slow freezing rates were found to be 0.45 and 0.35, respectively. The sample with a larger ice crystal forms a more porous structure during freeze‐drying. The higher drying rate of the sample with larger ice crystals can be attributed to the porous structure, which provides a path for vapor to escape, thereby enhancing the drying rate. Meanwhile, smaller ice crystals provide a hindrance to the vapor's escape, resulting in lower drying rates.

#### 3.4.2 Osmotic Predehydration (Nonbeneficial)

Drying time can be considerably reduced by using different pretreatments; this is quite well demonstrated for various food samples and drying methods. However, not many such works have been undertaken to explore different pretreatments for AFD. Only Donsi et al. and Rahman et al. have studied the effect of pretreatments on the drying rate of fresh shrimps. The fresh shrimps were dipped in an osmotic salt or sugar solution for 40 min or thermally vacuum‐dried. The osmotically pretreated shrimps experienced a reduced drying rate due to solid gain hindering moisture removal. On the flip side, thermal vacuum drying as a pretreatment showed higher drying rates (Donsì et al. 2001). Similarly, the study on various food products observed that the drying rate for osmotically pretreated samples was lower than that of untreated ones. Banana, carrot, and potato slices were dipped in sugar syrup, while beef meat, liver, and cod fish slices were dipped in salt solution for 30 min. The initial moisture content of the treated sample was lower than that of the untreated one, but the rate was affected negatively (Rahman et al. 2016). The negative effect of osmotic pretreatment was seen in both VFD and AFD. Another problem with the treatment is the depression of the freezing point due to an increase in the solute concentration. This requires drying at lower temperatures to keep the food in a frozen condition. The scanning electron microscopic images clearly depicted that the osmotic agents clogged the pores of the treated samples. This led to a decrease in the drying rate of the treated samples. Furthermore, the treated food sample exhibited poor color, rehydration ratio, and textural properties.

## 4 Energy Intensification of the System

The energy consumed in a process is pivotal in determining the feasibility of the process at an industrial scale. The shift of focus of both the industrialists and academia from VFD to AFD method was because of the lower energy consumption, owing to the elimination of the need for a vacuum in AFD. In addition, the AFD method also aligns well with the UN's Sustainable Development Goals (SGGs), particularly, SDG 7 (Affordable and Clean Energy), as it operates without a vacuum, thereby reducing energy demand; SDG 9 (Industry, Innovation and Infrastructure), as it enables innovation in low‐energy food and pharmaceutical drying technologies; and SDG 13 (Climate Action), as the technology demands relatively lesser energy input compared to VFD, thereby reducing the carbon footprint of the process. Likewise, the European Union's 2030 climate and sustainability goals, such as GHG emissions reduction, energy efficiency, and a sustainable food system, can also be achieved with this technology. Based on the reports in the literature, the AFD method has shown positive outcomes in the energy aspect and consumes reasonably less energy compared to VFD. A study demonstrated that AFD can help reduce energy costs by 35% compared to VFD (Wolff and Gibert 1990b). Based on a computational evaluation of energy consumption in AFD (fluidized bed with adsorbent and dehumidification by heating air), the study concluded that it can reduce the heating energy requirement by 9% and the cooling energy requirement by 37% compared to traditional VFD operations. Moreover, the same study also evaluated (computationally) a continuous operation for AFD and suggested it would further reduce the heating energy requirement by 38% and the cooling energy requirement by 34%. Similarly, a study using a desiccant dehumidification wheel for the generation of the cold, dry AFD air was undertaken, making a comparison with the energy consumption of VFD. For the conventional VFD process, electricity consumption by the condenser, vacuum pump, sublimation, and defrosting of ice at the end of the operation was considered. In contrast, in the case of AFD, energy is required for the refrigeration system to cool the air, for a blower or fan for continuous air circulation, and for heating or dehumidifying the air. The power demand for these associated enthalpies and operations is accounted to be 1.25 and 1.88–2.15 kWh/kg for a closed‐loop AFD and VFD, respectively (Chen et al. 2020). However, it is important to understand that the energy consumption, as well as the environmental impact of AFD, is highly dependent on the internal structure of the sample (Merone et al. 2020). For instance, the reduction in energy consumption for a cylindrical‐shaped sample versus a slab‐ or cuboidal‐shaped sample was 68.8% and 78.8%, respectively, when US‐assisted AFD was applied. It is also worth noting that applying any other method to intensify the process will further add to the energy consumption of the process. An increase in energy consumption as high as 163% per hour was seen when US, with 30.8 kW/m^3^, was applied during AFD at −10°C and 2 m/s. Despite that, energy consumption was reduced by 68.8%–78.8% (Moreno et al. 2017)

A comparative study by D. Xu et al. (2015) for air drying, VFD, and AFD at two different temperatures and temperature ramping was conducted. Air drying was carried out at 40°C, 1.5 m/s, and 20% RH; VFD at −40°C and 50 Pa; and AFD at −10°C, −5°C, and in a programmed temperature ramping experimental run with an air velocity of 1.5 m/s and 30% RH. The study revealed that the highest energy consumption was for VFD, followed by AFD at low temperature, AFD at high temperature, and AFD by the temperature ramping method, and lowest for the air‐drying method. Compared to VFD, AFD at −10°C showed 25.9% lower energy consumption, and increasing the temperature helped reduce the energy consumption by 33.9%. Adopting the temperature ramping strategy also helped reduce energy consumption by 37.1%.

## 5 Intellectual Property Rights and Commercialization

Table 5 gives the list of patent applications filed between 2000 and 2025. For reviewing all the appropriate patents related to the AFD process, a classification code F26B5/04 was used. The classification code suggests that the patents covered in this classification include inventions related to the drying of solid materials or objects by a process not involving heat application by evaporation or sublimation of moisture. Further, specific keywords, namely, “Atmospheric Freeze‐Drying,” “Freeze‐Drying in Atmospheric Pressure,” “Ambient Pressure Freeze‐Drying,” “Cold Air Freeze‐Drying,” and “Freeze‐Drying without Vacuum,” were used to find the relevant inventions related to the AFD process. Surprisingly, only 18 related inventions were filed between 2000 and 2025. Among these, eight applications are active, three have expired, five are pending, and one has withdrawn status. Moreover, within the active patents, seven are unique, and the rest are applied and granted for different regions. These data suggest that although much work is carried out at a laboratory scale for the AFD process, very few of these research investigations are made through an intellectual property right. The information on the current assignee of the granted patents suggests that the commercial companies hold the intellectual property rights of the patent; however, more information on the commercialization of the technology could not be traced back. This suggests a wide gap between the research and the commercialization of this technology, suggesting a poor implementation on an industrial scale. Furthermore, the applied patents can be further classified as spray‐based or non‐spray‐based depending on the process of AFD. The spray‐based AFD process is filed for liquid or suspension as a feed, whereas the non‐spray‐based process is applied for solid food materials.

**TABLE 5:** Filled patents on the AFD process between 2000 and 2025.

Patent no.	Title	Inventor	Assignee	Spray/non‐spray‐based	Status
US7007406B2	Powder formation by atmospheric spray freeze‐drying	Zhaolin Wang and Warren H. Finlay	Aerosol Therapeutics LLC	Spray based	Expired
JP5183068B2	Powder formation by atmospheric spray freeze‐drying	Warren H. Finlay and Zhaolin Wang	Aerosol Therapeutics LLC	Spray based	Expired
CA2450779C	Powder formation by atmospheric spray freeze‐drying	Warren H. Finlay and Zhaolin Wang	Aerosol Therapeutics LLC	Spray based	Expired
US8322046B2	Powder formation by atmospheric spray freeze‐drying	Zhaolin Wang and Warren H. Finlay	Aerosol Therapeutics LLC	Spray based	Active
WO2005061088A1	Powder formation by atmospheric spray freeze‐drying	Warren H. Finlay and Zhaolin Wang	Aerosol Therapeutics LLC	Spray based	Active
KR101512608B1	Process line for the production of freeze‐dried particles	Bernard Louis, Mathias Plitsko, Manfred Strusica	—	Spray based	Active
US9453676B2	Compositions and methods for atmospheric spray freeze‐drying	Thomas D. Robinson	Aerosol Therapeutics LLC	Spray based	Active
US9863701B2	Compositions and methods for atmospheric spray freeze‐drying	Thomas D. Robinson	Aerosol Therapeutics LLC	Spray based	Active
CN105992584B	Composition and method for atmospheric spray freeze‐drying	T. D. Robinson	Aerosol Therapeutics LLC	Spray based	Active
DE102017119649A1	Process for the continuous production of freeze‐dried droplets in a closed gas cycle	Anmelder Gleich	Individual	Spray based	Withdrawn
CN113280583A	Freeze‐drying method and apparatus	Wang Zhaolin, Qian Hua, Wang Qijun	Nanjing Zhiyikaiwu Technology Co., Ltd.	Non‐spray based	Active
CN112503863A	Normal pressure freeze‐drying equipment	Xiang Junhui, Yu Xianbo, Xiao Zhou	Youpeng Jiaxing New Materials Technology Co ltd	Non‐spray based	Pending
CN115342603A	Circulating air freeze‐drying system and method	Gong Maoqiong, Liu Ying, Zhao Yanxing, Wang Haocheng, Guo Hao	Technical Institute of Physics and Chemistry of CAS	Non‐spray based	Pending
CN115451663B	Freeze‐drying system and method for adsorption dehydration by using circulating air	Gong Maoqiong, Liu Ying, Zhao Yanxing, Wang Haocheng, Guo Hao	Technical Institute of Physics and Chemistry of CAS	Non‐spray based	Active
CN117414684A	Compressed air freezing dryer and use method thereof	Zheng Chaomin, Yang Wenbing, Song Xin, Tong Weizhong	Zhejiang Ruizong Electromechanical Equipment Co., Ltd	Non‐spray based	Pending
US6584782B2	Method for producing particulate goods	Hans Leuenberger, Armin Karl, Theodor Prasch, Bernhard Luy	Glatt GmbH	Spray based	Expired
US20200149816A1	Apparatus and method for continuous lyophilization	Edward Weisselberg	KOMLINE–SANDERSON CORPORATION Wyssmont Co Inc Komline Sanderson Corp	Non‐spray based	Active
CN101189980A	Method for using heat pump atmospheric freeze‐drying to prepare dehydrated fruits and vegetables	Li Sheng	Individual	Non‐spray based	Pending

*Note*: Data retrieved on March 5, 2025.

## 6 Recommendation for Future Research

The acceleration methodologies for AFD appear promising in improving the drying time. However, some of these methodologies are still underexplored and can provide a beneficial effect. For instance, IR heating can provide significant benefits as an acceleration method specifically for AFD, but has not been systematically investigated. Since AFD is fundamentally an internal diffusion‐limited process, future work should prioritise methods that enhance internal diffusion rather than solely focusing on extrinsic factors. Additionally, structural modifications of the sample to reduce internal diffusion are an entirely unexplored research area in the context of AFD. This could be explored for the products where the final shape is not important, especially for powdered final products. Finally, efforts should be directed toward strategies that enable an easier scale‐up for AFD and make it available as a viable alternative to VFD. Likewise, a systematic comparison of VFD and AFD in terms of initial as well as operating costs, along with the acceleration method employed (with AFD) under equivalent conditions, is still lacking and would provide better insights into the economic viability of AFD.

## 7 Conclusion

The AFD process can potentially reduce the cost and energy consumption compared to the conventional lyophilization method. However, the lower sublimation rates increase the process time. The processing time for AFD is consistently higher than VFD in most cases. The acceleration of AFD with different techniques is quite promising in improving drying rates. Simple methods, such as temperature ramping, size reduction, and changes in the shape of the sample, have been shown to largely alleviate the drying rates. MW, US, and fluidization technologies also improved the rates. However, the deterioration of the quality characteristics, such as the colour and texture, is inevitable. Additionally, they can increase the processing cost and energy consumption. The use of adsorbents appears promising, but compatibility issues and additional operational steps for removal and regeneration of the adsorbent are required. Studies pertaining to the pretreatment of the sample for AFD are quite limited and largely unsuccessful. A few emerging methods, such as impinging jets and various preparatory approaches to enhance the drying rate, are encouraging. There is a lack of commercialization of the applied method at the laboratory scale. Future research should focus on improving the rate of internal diffusion and conducting a systematic comparison between AFD and VFD in terms of economic feasibility to establish AFD as a viable alternative.

## Author Contributions

**Nikita S. Bhatkar**: conceptualization, data curation, investigation, methodology, writing – review and editing, writing – original draft. **Anarghya Murthy**: conceptualization, writing – review and editing, supervision. **Siew Young Quek**: conceptualization, supervision, writing – review and editing. **Meng Wai Woo**: conceptualization, supervision, visualization, writing – review and editing.

## Conflicts of Interest

The authors declare no conflicts of interest.
